# Bacteriophage Cocktails Protect Dairy Cows Against Mastitis Caused By Drug Resistant *Escherichia coli* Infection

**DOI:** 10.3389/fcimb.2021.690377

**Published:** 2021-06-17

**Authors:** Mengting Guo, Ya Gao, Yibing Xue, Yuanping Liu, Xiaoyan Zeng, Yuqiang Cheng, Jingjiao Ma, Hengan Wang, Jianhe Sun, Zhaofei Wang, Yaxian Yan

**Affiliations:** School of Agriculture and Biology, Shanghai Jiao Tong University, Shanghai Key Laboratory of Veterinary Biotechnology, Shanghai, China

**Keywords:** cow mastitis, *Escherichia coli*, phage cocktails, therapy, drug resistant

## Abstract

Mastitis caused by *Escherichia coli* (*E. coli*) remains a threat to dairy animals and impacts animal welfare and causes great economic loss. Furthermore, antibiotic resistance and the lagged development of novel antibacterial drugs greatly challenge the livestock industry. Phage therapy has regained attention. In this study, three lytic phages, termed vB_EcoM_SYGD1 (SYGD1), vB_EcoP_SYGE1 (SYGE1), and vB_EcoM_SYGMH1 (SYGMH1), were isolated from sewage of dairy farm. The three phages showed a broad host range and high bacteriolytic efficiency against *E. coli* from different sources. Genome sequence and transmission electron microscope analysis revealed that SYGD1 and SYGMH1 belong to the *Myoviridae*, and SYGE1 belong to the *Autographiviridae* of the order *Caudovirales*. All three phages remained stable under a wide range of temperatures or pH and were almost unaffected in chloroform. Specially, a mastitis infected cow model, which challenged by a drug resistant *E. coli*, was used to evaluate the efficacy of phages. The results showed that the cocktails consists of three phages significantly reduced the number of bacteria, somatic cells, and inflammatory factors, alleviated the symptoms of mastitis in cattle, and achieved the same effect as antibiotic treatment. Overall, our study demonstrated that phage cocktail may be a promising alternative therapy against mastitis caused by drug resistant *E. coli*.

## Introduction

Mastitis (intramammary inflammation) remains a devastating disease in dairy animals worldwide. It adversely threatens the health of udder, decreases the quality and production of milk, impedes the growth of bovine, increases rearing and prevention costs, and negatively impacts animal welfare (Khan et al., 2021). The incidence of clinical mastitis in China from 2015 to 2017 was estimated to range between 0.6% and 18.2% monthly among seven farms, which cost 12000 to 76000 USD/farm/month (He et al., 2020).

Mastitis usually caused by *Escherichia coli* (*E. coli*), *Streptococcus uberis*, *Staphylococcus aureus*, and *Klebsiella pneumonia* (Bar et al., 2008; Klaas and Zadoks, 2017; Machado and Bicalho, 2018). Bacterial culture was done on milk samples from 161 large Chinese (>500 cows) dairy farms, the most frequently isolated pathogens were *Escherichia coli* (14.4%), *Klebsiella* spp. (13.0%), coagulase-negative staphylococci (11.3%), *Streptococcus dysgalactiae* (10.5%), and *Staphylococcus aureus* (10.2%) (Gao et al., 2017). Among the pathogens, *E. coli* is the most common Gram-negative bacteria causing acute clinical mastitis in dairy cows during early lactation (Cortinhas et al., 2016). During *E. coli* mastitis, the common visible symptoms of the affected part include the complete udder showing redness, swelling, pain upon touch, and the highest loss of milk or abnormal milk. Also, the cows become anorexic and pyrexial, thereby resulting in low production of milk (Khatun et al., 2013b). Additionally, *E.coli* mastitis incurs subclinical phenotypes, including an increase in somatic cell count (SCC) in milk (Abdi et al., 2021) and the production of proinflammatory cytokines in blood (Cobirka et al., 2020). Currently, several measures to control mastitis have been taken, such as antibiotic therapy (Mcdougall et al., 2019), antimicrobial peptides (Gurao et al., 2017), bacteriophage therapy (Ngassam-Tchamba et al., 2020), probiotics (Pellegrino et al., 2018), and nanoparticle-based therapy (Orellano et al., 2019). Among them, because of their remarkable effectiveness, antibiotics are the most common method (Gomes and Henriques, 2016). However, due to the abuse of antibiotics, multidrug-resistance bacteria have emerged which is projected to be the greatest challenges of the 21^st^ century (Streicher, 2021).

Bacteriophages (referred to hereafter as phages) are a group of viruses that can infect and kill bacteria at the end of their lytic cycle (Rehman et al., 2019). Phages and their bacterial hosts coexist and coevolve in various environments, such as soil, oceans, freshwater, humans, and animals (Zalewska-Pitek and Pitek, 2020). Phages are the most abundant entities on earth, with approximately 10^31^ individual particles, which outnumber their hosts by 10 to 100 fold, depending on the environment, seemingly keeping an endless equilibrium state (Moelling et al., 2018; Zalewska-Pitek and Pitek, 2020). Further, interest in phage therapy began when a ‘bacteriolytic agent’ was discovered, and phage treatments were quickly applied to humans at the beginning of the 20^th^ century (Chanishvili, 2012). Phage preparations remain in use and commercially available today in many countries, such as Georgia and Russia (Kutter et al., 2010). Although little progress has been made in phage research, phage therapy has been renewed due to the threat of multidrug-resistant bacterial infection and the advantages of phages, including specificity, efficacy, harmlessness to humans, and diversity (Clara, 2018; Aslam and Schooley, 2019). To increase efficacy, phages can be used alone, as cocktails, or synergistically with other antimicrobials *in vitro* or *in vivo* (Melo et al., 2020). Unprecedentedly, researchers have used a cocktail of three engineered phages to a 15-year-old patient who developed cystic fibrosis with *Mycobacterium abscess* infection, and the patient’s clinical conditions improved without adverse reactions (Dedrick et al., 2019). A 63-year-old female patient suffering recurrent urinary tract infection (UTI) caused by drug-resistant *Klebsiella pneumoniae* (ERKp) was treated with antibiotic and phage cocktail. After treatment, the patient successfully combated UTI and was cured (Bao et al., 2020). Hence, phage therapy is an attractive approach for treating multidrug-resistant organisms.

To evaluate the efficiency and potential of the phage cocktail in treating cow mastitis, we isolated *E.coli* phages from dairy farm sewage. To learn more details, we further tested the spectra, efficiency of plating (EOP), and biological properties of three phages. We also identified their morphology and sequenced their genome. Finally, we evaluated their potential as phage therapy candidates in cows with mastitis caused by *E.coli* infection. This study expands our knowledge of the phage genome and highlights that phages have great potential as therapeutic adjuncts to relieve the embarrassing situation of antibiotic resistance.

## Materials and Methods

### Ethics Statement

The animal experiments were carried out according to animal welfare standards and approved by the Ethical Committee for Animal Experiments of Shanghai Jiao Tong University, China. All animal experiments complied with the guidelines of the Animal Welfare Council of China.

### Bacterial Strains and Culture Conditions

Eighteen *E. coli* (12 Food-borne strains and six strains isolated from the milk of dairy cows with mastitis) and two *E. coli* reference strains of MC1061 and MG1655 from the American Type Culture Collection (ATCC) were used in this study were stored in our laboratory (**Table 1**). Bacteria were cultured at 37°C in Luria-Bertani (LB) broth in a shaker at 200 rpm. The double-layer plate for purifying the phages was prepared by solid media containing LB and soft agar overlays, containing 0.75% agar into the LB broth agar (1.5%). Also, 5% sheep blood agar containing ampicillin (100 μg/mL) was used to determine the number of CFU/mL in milk sample.

**Table 1:** The host range and EOP of vB_EcoM_SYGD1, vB_EcoP_SYGE1, and vB_EcoM_SYGMH1.

Bacterial No.	Source^a^	vB_EcoM_SYGD1		vB_EcoM_SYGE1		vB_EcoM_SYGMH1	
		Spot test^b^	EOP^c^	Spot test	EOP	Spot test	EOP
**MG1655**	I	—	—	++	0.79 ± 0.03	—	—
**MC1061**	I	++	1.00	++	1.00	++	1.00
**DG03512**	II	++	0.66 ± 0.03	+	0.07 ± 0.00	++	0.20 ± 0.02
**Min27**	II	++	0.94 ± 0.02	++	0.40 ± 0.02	++	1.19 ± 0.02
**ECS1**	II	+	0.10 ± 0.01	+	0.11 ± 0.01	++	0.34 ± 0.02
**ECS2**	II	—	—	—	—	—	—
**ECS3**	II	++	0.36 ± 0.02	++	0.26 ± 0.01	+	0.09 ± 0.01
**ECS4**	II	—	—	—	—	—	—
**ECS5**	II	++	0.29 ± 0.02	++	0.29 ± 0.02	++	0.31 ± 0.02
**ECS6**	II	++	0.27 ± 0.03	++	0.30 ± 0.02	++	0.21 ± 0.02
**ECS7**	II	+	0.02 ± 0.00	+	0.21 ± 0.02	++	0.30 ± 0.02
**ECS8**	II	++	0.74 ± 0.03	++	0.41 ± 0.01	++	0.49 ± 0.03
**ECS9**	II	++	0.48 ± 0.04	++	0.28 ± 0.01	++	0.26 ± 0.00
**ECS10**	II	—	—	++	0.33 ± 0.02	—	—
**ECD1**	III	+	0.11 ± 0.02	+	0.19 ± 0.02	+	0.24 ± 0.02
**ECD2**	III	++	0.42 ± 0.02	++	0.49 ± 0.01	++	0.62 ± 0.06
**ECD3**	III	—	—	+	0.21 ± 0.02	—	—
**ECD4**	III	—	—	+	0.17 ± 0.01	—	—
**ECD5**	III	—	—	—	—	—	—
**ECD6**	III	—	—	—	—	—	—

a I, purchased from American Type Culture Collection; II, hospital-acquired strains; III, clinically-isolated strains from the milk of dairy cows with mastitis.

b ++, clear plaque; +, hazy plaque, and -, no plaque.

c EOP, efficiency of plating (EOP = phage titre on test bacterium/phage titre on strains MC1061). The EOP values are shown as the mean of the three repeats ± SD. -, no plaque on target bacterium.

### Antibiotic Susceptibility Testing

All strains used in this study were subjected to antibiotic susceptibility testing against ampicillin, cefepime, gentamicin, kanamycin, ciprofloxacin, norfloxacin, and meropenem using the Kirby Bauer disc diffusion method. Briefly, bacteria were grown to an optical density at 600 nm (OD_600_) of 0.4 to 0.6 in 3 mL LB, and 200 μL of each culture was swabbed on the surface of LB agar plates. Antibiotic discs (Beijing Pronade technology co., LTD, Beijing, China) were placed on the swabbed culture incubated for 16 to 18 h at 37°C, following which the inhibition zone was measured, and each strain was determined as resistant/intermediate/sensitive to each antibiotic tested following the chart provided by the manufacturer.

### Isolation of Phages

Phages were isolated from the sewage of the different dairy farms in Shanghai using a traditional method, as described previously, with some modifications (Wang et al., 2016). Briefly, all samples were centrifuged at 5,000 g for 20 min at 4°C, and the debris of supernatants was removed through the 0.22 μm microporous membrane and stored at 4°C. The pre-filtered samples were co-cultured with logarithmic phase DG03512, MG1655, and Min27 host strains for 12 h at 37°C before re-filtering through a 0.22 μm membrane filter to discard bacteria and their debris. The enriched phage suspensions were mixed with host strains (mid-log phase, OD_600_ = 0.4 to 0.6), and the mixture was added to 10 mL soft agar (0.75% agar) before pouring on top of an LB agar plate (1.5% agar). The plates were cultured at 37°C for 12 h until plaques were observed. To gain pure phage lysate, a single plaque was picked, and the above process was repeated following three successive times purification. Finally, phages were stored at 4°C or −80°C.

Phage particles were concentrated and purified by polyethylene glycol (PEG) precipitation and CsCl step gradients (Baker et al., 2006). Briefly, 200 mL of exponential-phase indicator bacteria was infected with phage stocks for 5 h at 37°C with shaking, and the supernatant lysates were collected by centrifuging at 10,000 g for 10 min before filtering through a 0.22 µM filter. To precipitate the phage particles, PEG 8000 was added at 10% final concentration to the supernatant, the mixture was incubated overnight at 4°C with constant shaking before centrifuging at 10000 g for 20 min, and the supernatant was removed. The resulting pellets were re-suspended in SM buffer (100 mM NaCl, 10 mM MgSO_4_·7H_2_O, and 50 mM Tris·HCl pH 7.5). After the addition of 0.5 g/mL CsCl, the mixture was layered on top of CsCl step gradients (densities of 1.15 1.45, 1.50, and 1.70 g/mL) in Ultra-Clear centrifugation tubes and centrifuged at 28,000 × g for 2 h at 4°C, dialyzed in SM buffer. Phages were stored at 4°C or −80 °C for further experiments.

### Determination of the Lytic Activity of the Phages Against Clinical Isolates of *E. coli*

The infection and lysis capacity of phages were determined using the spot test method, as previously described (Shahin et al., 2020). Briefly, 100 µL of 20 exponential growth strains were mixed evenly with 10 mL semisolid LB medium and overlaid on the LB Agar (1.5% agar). Also 5 µL of undiluted phage suspensions (~10^7^ PFU/mL) were dropped on the culture surface. The plates were incubated at 37°C overnight, and a clear zone at the spot area indicated bacterial susceptibility to the phage. EOP, the ratio of the phage titer on the test strain to the phage titer obtained from the reference bacteria, was performed using the double-layer agar method. The reference strains for all isolated phages were MC1061. The experiment was performed in triplicate, and the results were recorded as the EOP mean ± standard deviation (SD). Finally, the EOP of each phage was classified as high (EOP ≥ 0.5), medium (0.1 ≤ EOP ≤ 0.5), low (0.001 < EOP < 0.1), and no lysis (EOP ≤ 0.001) (Shahin et al., 2021).

### DNA Extraction and Sequencing

Phage DNA was extracted from 10 mL concentrated phage suspensions (10^10^ PFU mL^−1^) using phenol-chloroform extraction and ethanol precipitation methods (Wang et al., 2016). Subsequently, Illumina MiSeq system was used for phages whole genome analysis. Sequence alignments were carried out using the Accelrys DS Gene software package of Accelrys Inc. (USA). Putative open reading frames were suggested using the algorithms of the software packages Accelrys Gene v2.5 (Accelrys Inc.) and ORF Finder (NCBI). Identity values were calculated using different BLAST algorithms (http://www.ncbi.nlm.nih.gov/BLAST/) at the NCBI homepage. The sequences of phages were submitted to the NCBI. tRNA coding regions were searched using tRNAscan-SE 2.0 (Lowe and Chan, 2016).

### Morphological Characterization

Purified phage particles (10^9^ PFU/mL) were placed onto carbon-coated copper grids and negatively stained with 2% (w/v) uranyl acetate (pH 6.7) for 10 min. Finally, the purified viral particle morphology was examined using a FEI TEM Tecnai G2 Spirit Biotwin (FEI, Hillsboro, US) at an accelerating voltage of 120 kV.

### One-Step Growth Curve

The one-step growth curves of phages were determined as previously reported (Yang Z. et al., 2019). Briefly, mid-exponential phase *E. coli* DG03512, MG1655, and Min27 were infected with vB_EcoM_SYGD1, vB_EcoP_SYGE1, and vB_EcoM_SYGMH1 at a multiplicity of infection (MOI) of 10, 1, 0.1, 0.01, 0.001, and 0.0001, respectively. After incubation for 4 h, the optimal MOIs of SYGD1, SYGE1, and SYGMH1 were determined using the double-layer agar method. For one-step growth experiments, host cells grew to an exponential phase and were infected with phages at the optimal MOI. After absorption for 10 min at 37°C, the cultures were centrifuged for 1 min at 13000 g to remove the unabsorbed phages. The pellets were washed with an LB medium, followed by resuspension in an equivalent LB medium. The cultures were grown at 37°C with shaking at 160 rpm. Samples were taken every 10 min (up to 120 min), and the number of phage particles was quantified using the method described above. The LB medium was only used as a control, and all experiments were conducted in triplicate.

### Biological Characteristics of Phages

The procedures were performed as described previously, with modifications (Lu et al., 2017). To determine the thermostability of the three phages, phage suspensions at 10^8^ PFU/mL were subjected to different temperatures (25°C, 37°C, 45°C, 50°C, 60°C, and 70°C) for 1 h. To evaluate the stability of the phage at different pH levels, phage suspensions were incubated in an LB broth adjusted to pH values ranging from 2 to 12 with HCl or NaOH for 1 h. Additionally, the ultraviolet (UV) sensitivity of phages was determined by exposing the phage suspensions at 35 cm under a UV light (30 w), and 100 μL aliquots were collected every 15 min until 75 min. The sensitivity of phages to chloroform was examined by mixing phage suspensions with different concentrations (5%, 25%, 50%, and 75%) of chloroform, respectively, and shaking vigorously, then incubated at 37°C for 30 min. The mixtures were centrifuged at 800 rpm for 15 min, and the hydrophilic layer was collected. The phage suspensions were diluted serially with 1 × SM buffer, and phage titers were calculated using double-layer agar methods. All tests were performed in triplicate.

### Determination of Endotoxins of Phage Cocktails

To improve the safety of the phage, an affinity matrix of modified polymyxin B (PMB) (GenScript, Nanjing, China) was used to remove phage cocktail endotoxins. Furthermore, the endotoxin levels of the phage cocktails were evaluated by colorimetric method following the recommendations of the manufacturer (GenScript). The end-product was measured spectrophotometrically in a microplate reader.

### The Treatment of Cow Mastitis Caused by *E. Coli* Using Phage Cocktails

The antibacterial activity of phage cocktails was evaluated in cows. We selected eight milk-secreting Holstein heifers (4–5 years old, 3–5 months after calving), and they were randomly divided into four groups, with two animals each. Three groups of cows were intramammary challenged with 60 CFU ECD2 suspended in 1 mL of pyrogen-free phosphate buffer saline PBS (Khatun et al., 2013a). After 24 h of inoculating *E. coli*, milk and blood samples were collected and analyzed immediately. Phage cocktails containing SYGD1, SYGE1, and SYGMH1 were prepared by mixing the three phages at a 1:1:1 ratio with a primary concentration of about 10^10^ PFU/mL, respectively. Finally, the mixture was diluted 100 times using (PBS) for therapy. One group was intramammary treated with 5 mL ceftiofur sodium (600 mg/mL). The second group was intramammary injected with 5 mL phage cocktails (1 × 10^8^ PFU/mL). The third group was intramammary treated with 5 mL PBS alone. All treatments were administered once a day for three consecutive days. The fourth group, as a control group, was neither challenged nor treated. The eight animals were kept separately during the trial and monitored every day. After three-day treatment, milk and blood samples were collected and detected for three consecutive days.

### Bacteriological Loading and Somatic Cell Count (SCC)

Milk samples were collected at 0 day before treatment and 4, 5, and 6 days after the beginning of the treatment from each individual quarter into 50 mL sterile tubes. Serial 10-fold dilutions of each milk sample were made under aseptic conditions by pipetting the sample into sterile PBS, and three 10 μL drops were plated on 5% sheep blood agar containing 100 μg/mL of ampicillin because of the resistance of ECD2 to ampicillin, and count colony was started after 24-hour incubation at 37°C. SCC of each milk sample was determined using a cell counter immediately after collection. Briefly, 10 μL of diluted fresh milk samples were thoroughly mixed with Trypan Blue and spread on a cell-counting plate.

### Determination of Inflammatory Cytokine Levels in Serum

Blood samples were collected on the sixth day in 9 mL blood tubes stabilized with 50 to 200 IU of Na-heparin and immediately placed on ice for inflammatory biomarker analysis. A hot water bath (50°C) was used to evaporate the extracted solution. The blood tubes were centrifuged at 2,000 g at 4°C for 20 min. Serum was collected and stored at −20°C until used. Concentrations of inflammatory biomarker interleukin (IL)-1β and tumor necrosis factor (TNF)-α were measured *via* an enzyme-linked immunosorbent assay kit (ELISA kit) (Abcam, England) following the manufacturer’s recommendations.

### Statistical Analysis

All statistical analyses, unless otherwise stated, were executed using GraphPad Prism 8 (Graph Pad Software, Inc., La Jolla, CA). Error bars in graphs show the standard error of the mean (± SEM). The unpaired t-test was applied to compare the differences between each two groups. Most assays were performed in triplicate, and the data were expressed as the mean of three independent experiments. Statistical significance was considered as *p* < 0.05.

### Data Availability

The whole-genome sequences of vB_EcoM_SYGD1, vB_EcoP_SYGE1, and vB_EcoM_SYGMH1 can be found in the GenBank Access Code MW883059, MW883060 and MW883061.

## Results

### Phage Isolation and Host Range Determination

In this experiment, three samples from different dairy farms in Shanghai were detected to isolate the phages. Using DG03512, MG1655, and Min27 as host strains, we successfully isolated three phages that could form clear plaques in the lawn of their host cells, and phages were named vB_EcoM_SYGD1, vB_EcoP_SYGE1, and vB_EcoM_SYGMH1, respectively. Spot assay showed that SYGD1 and SYGMH1 had a wide host range with a similar spectrum (12 of 20, 60%), and SYGE1 could lyse more strains (16 of 20, 80%) (**Table 1**). In particular, 90% strains were resistant to two or more antibiotics (**Table 2**). EOPs assay (**Table 1**) revealed that SYGE1 had higher production levels of progeny phages compared to SYGD1 and SYGMH1. The high and medium EOPs levels that is, the ratio of titers ≥ 0.1, of the former phage were observed in about 93.8% of tested strains; SYGD1 and SYGMH1 was 91.2% (11 out of 12 strains).

**Table 2:** Antibiotic susceptibility profiles of *E. coli* strains used in this study.

Strains	AMP	FEP	GEN	KM	CIP	NOR	MEM
MG1655							
MC1061							
DG03512							
Min27							
ECS1							
ECS2							
ECS3							
ECS4							
ECS5							
ECS6							
ECS7							
ECS8							
ECS9							
ECS10							
ECD1							
ECD2							
ECD3							
ECD4							
ECD5							
ECD6							

	*Resistant.*	
	*Intermediate.*	
	*Sensitive.*	

AMP, Ampicillin; FEP, Cefepime; GEN, Gentamicin; KM, Kanamycin; CIP, Ciprofloxacin; NOR, Norfloxacin; MEM, Meropenem.

### Phage Morphology and Characteristics of the Phage Genome

**Figure 1:**
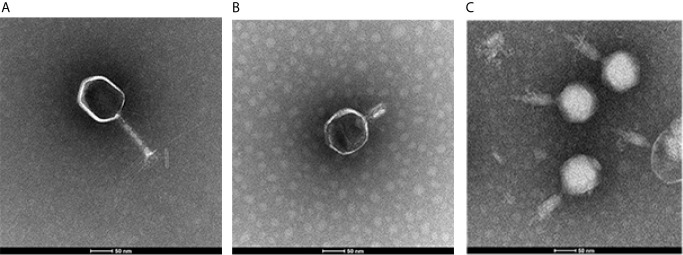
Images of phage particles by transmission electron microscopy. **(A)** vB_EcoM_SYGD1. **(B)** vB_EcoP_SYGE1. **(C)** vB_EcoM_SYGMH1 (scale bar was 50 nm).

**Figure 2:**
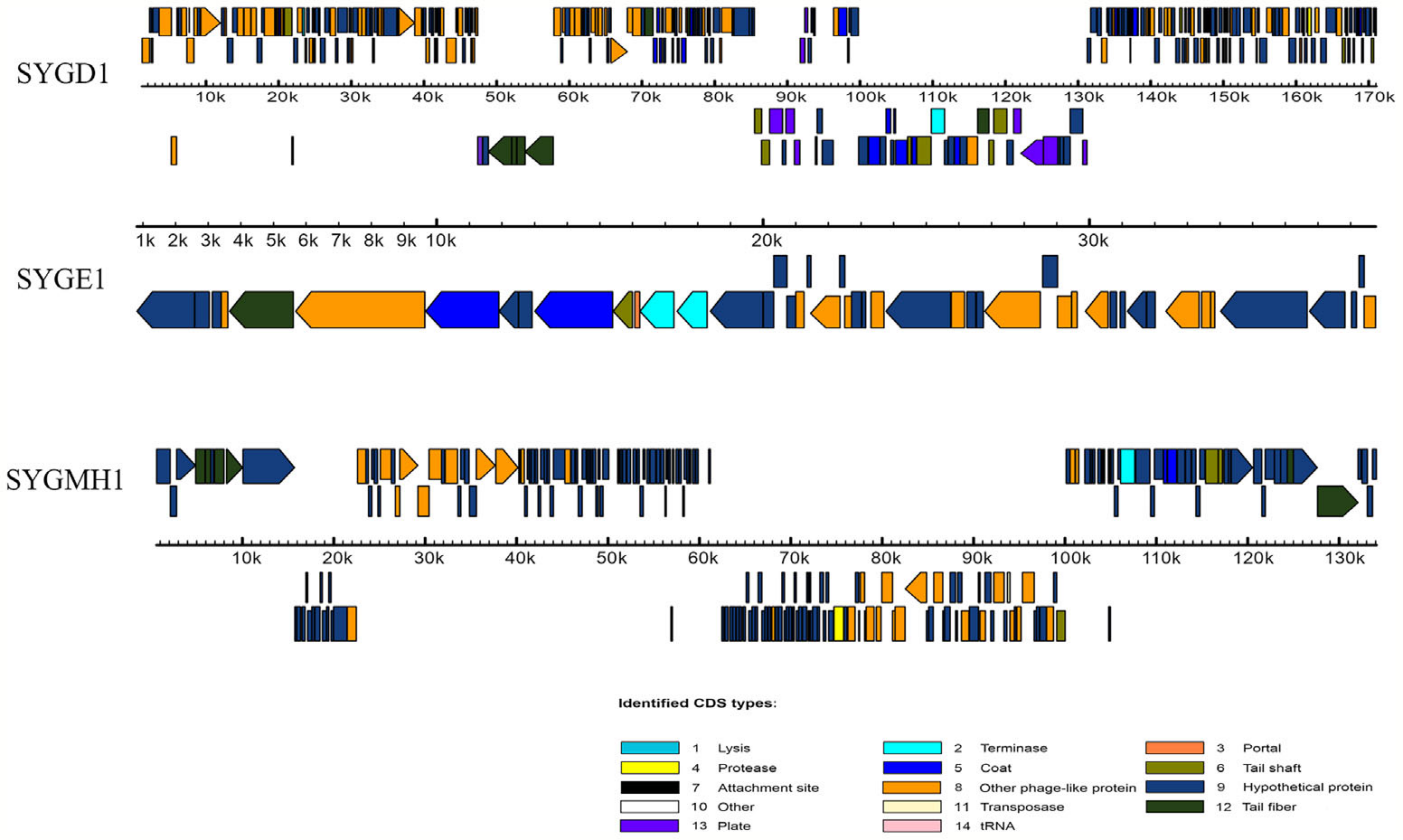
Genome features of vB_EcoM_SYGD1, vB_EcoP_SYGE1 and vB_EcoM_SYGMH1. Their predicated ORFs and their orientations are represented by arrows. The function modules are shown in different colors.

To determine the morphological characteristics of phages, the purified viral particles were stained, and TEM was performed. As shown in **Figure 1**, SYGD1 (**Figure 1A**) and SYGMH1 (**Figure 1C**) displayed a highly similar appearance with a prolate icosahedral head about 50–60 nm in diameter and a long tail with a length of nearly 100 nm and a width of about 10 nm surrounded by a helical sheath and long-tail fibers attached to the baseplates, similar to the *Myoviridae* family *o*f the order *Caudovirales*. Phage SYGE1 (**Figure 1B**) was smaller than the other two phages with a short tail of about 10 nm, and had a head diameter of 30–40 nm which was typical for *Autographiviridae* phages. Genome sequencing analysis revealed that SYGD1, SYGE1, and SYGMH1 comprised 171.3 kb, 39.7 kb, and 137.4 kb, with G + C content of 35.33%, 48.69%, and 43.56%, as well as 271, 46, and 205 proposed open reading frames (ORFs), respectively. There were 8 and 6 tRNAs in the genome of SYGD1 and SYGMH1, respectively. No ORFs encoding tRNA were found in SYGE1 by analysis with tRNAscan-SE 2.0 (details were shown in **Supplementary Tables S4, S5**). The genome of phage SYGE1 were flanked with terminal repeats with a size of 170 bp. All of them comprised a typical modular format, including DNA replication and modification, structural components and DNA packaging, tail structural components and host cell lysis (**Figure 2**). Furthermore, the BLAST analysis revealed that SYGD1 and SYGMH1 belonged to T4-like phage (**Table 2**), which showed relatively high homology (about 95%–97%) to NCBI database published T4-like phage (**Supplementary Tables S1, S3**). SYGE1 belonged to a T7-like phage, which showed partial homology (about 95%) to published T7-like phage (**Supplementary Table S2**). There are no homologs of known harmful genes, such as virulence, antibiotic resistance genes, or lysogenic genes, in the genome of phages. Thus, SYGD1, SYGE1, and SYGMH1 were considered lytic phages.

### Determination of One-Step Growth Curve

**Figure 3:**
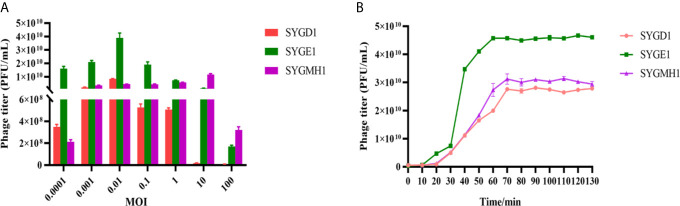
The optimal MOI and one-step growth curve of phages. **(A)** vB_EcoM_SYGD1, vB_EcoP_SYGE1, and vB_EcoM_SYGMH1 infected their host strain at an MOI of 0.01 or 10, reaching their peak titer, indicating the most suitable concentration for lysing bacteria. **(B)** SYGD1, SYGE1, and SYGMH1 infected their host strains at the optimal MOI. The supernatants were harvested at 10 min intervals post-infection, and titers were determined using the double-layer method.

From **Figure 3A**, SYGD1 and SYGE1 infected their host strains at an MOI of 0.01, generating the highest phage titer, while the SYGMH1 optimal MOI was 10. From **Figure 3B**, the one-step growth curve of SYGD1 and SYGMH1 had similar characteristics, that both of them had latent period of about 20 min with almost no release of progeny phages and entered about 50 min and 70 min lysis period with a burst size of 52 and 58 PFU/cell, respectively. SYGE1 exhibited a relatively shorter latent period was about 10 min and the burst size was estimated as 129 PFU per infected cell.

### Biological Characteristics of Phages

**Figure 4:**
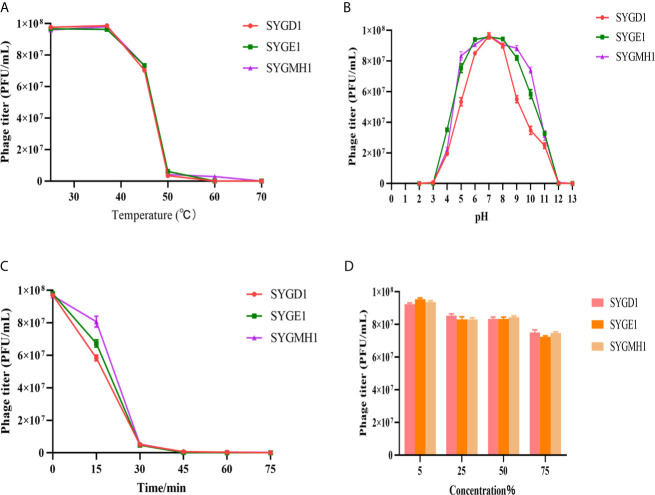
Stability of vB_EcoM_SYGD1, vB_EcoP_SYGE1, and vB_EcoM_SYGMH1 under various conditions. **(A)** Temperature. **(B)** pH stability. **(C)** UV radiation stability. **(D)** Chloroform sensitivity. Survived phage particles were determined by double-layer tests. Error bars show the SEM among triplicate samples.

To examine the characteristics of the three phages, we analyzed their sensitivity to temperature, pH, UV, and chloroform. From **Figure 4A**, SYGD1, SYGE1, and SYGMH1 had similar thermostability under 25°C–37°C; with an increase in temperature, the activity of phage decreased gradually. When the temperature reached 60°C, the phage titer decreased significantly, especially SYGD1, with only 3.4% survival. The activity of the three phages was completely lost after incubation at 70°C. Comparing, higher temperature inactivated the phages, but SYGMH1 had a relatively good resistance to temperature than other two phages. The pH sensitivity test showed that the three phages survived over a broad pH range (3–10), and pH 5–9 was their optimal growth condition, but became inactivated when pH was below 5 or above 10, indicating that the three phages were sensitive to strong acid or alkali (**Figure 4B**). From **Figure 4C**, all the phages were sensitive to UV radiation, and their activity decreased sharply after short exposure. Although the chloroform sensitivity test suggested that the activity of phages decreased with the prolongation of incubation time or the increase in chloroform concentration, it was not significant, indicating that phages were tolerant of chloroform (**Figure 4D**).

### Phage Therapy Alleviates CFU Loads and Inflammatory Response *In Vivo*

**Figure 5:**
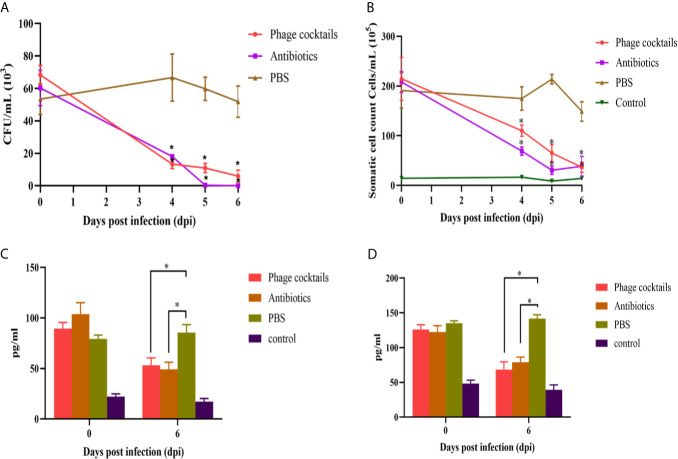
Antibacterial efficacy of phage cocktails against mastitis induced by *E.coli*. Cows challenged by *E.coli*-induced mastitis were intramammarily infused with phage cocktails containing vB_EcoM_SYGD1, vB_EcoP_SYGE1, and vB_EcoM_SYGMH1, and the bacteria load **(A)** and SCC **(B)** of milk samples were detected after three-day treatment, every 24 h. Concentrations of IL-1β **(C)** and TNF-α **(D)** in the sera of cows before treatment (0 day) and on the sixth day after beginning of treatment were measured using indirect ELISA. Data were analyzed using GraphPad Prism v 8.0 software. *Indicate significant differences between phage cocktails or antibiotics and untreated groups, as calculated by *t*-tests (*p* < 0.05).

The lipopolysaccharide of Gram-negative bacteria is always recognized by host cell receptors (such as TLR-4), which is one of the induced factors of pathogenesis for mastitis (Griesbeck-Zilch et al., 2008; Bhattarai et al., 2018). The endotoxins of crude phages were higher up to 8 EUs/mL. After CsCl purification, the endotoxins were reduced the amounts to 0.48 EUs/mL (data not shown), which is a safe level for animal experiments (Bonilla and Barr, 2018; Luong et al., 2020). Cows were intramammary infused with phage cocktails or antibiotics for three consecutive days after infection. From **Figure 5A**, PBS-treated group showed high bacterial loads at 4 day after infection, approximately 6 × 10^4^ CFU/mL. However, compared to the untreated groups, both phage-treated and antibiotic-treated groups showed statistically significant decreases in CFU burden at all-time points. On the sixth day, the antibiotic or phage-treated groups almost could not detect bacteria. Milk SCCs were estimated once collected. High SCC values indicated a high prevalence of subclinical mastitis. From **Figure 5B**, cows treated with phage cocktails and antibiotics had lower SCC values than untreated cows. On the sixth day, the SCC values of the treated groups were basically the same as those of the healthy groups after three-day treatment. These results demonstrate that the quality of milk is efficiently enhanced through phage therapy. Analysis of cytokines showed that cows with mastitis significantly increased the levels of IL-1β and TNF-α. The cytokine in the phage and antibiotic-treated groups revealed a pattern of decreased proinflammatory markers after three-day treatment, indicating that phage therapy can alleviate inflammatory reaction (**Figures 5C, D**).

## Discussion

Recently, *E. coli* has become the most important pathogen inducing cow mastitis, mainly causing high fever or lower milk production, even resulting in lethal consequences (Fazel et al., 2019; Guerra et al., 2020). Also, *E. coli* is an important antibiotic-resistant priority pathogen and can disseminate resistant genes in a process known as ‘horizontal transfer’ into other microbial communities (Jianying et al., 2008). Therefore, with the increase in antibacterial-resistant bacteria and discharge of antibiotics to the environment prompted scientists to explore alternative treatments.

Phage therapy is currently considered an efficient and suitable replacement for antibiotic therapy due to its specificity, safety, productive potential and lower economic burden (Mathieu et al., 2019; D'Accolti et al., 2021). Environmental source contains abundant phages which are able to lyse their target and the successful phage isolation is greatly facilitating the understanding of the ecology and the development of phage therapy (Gill and Hyman, 2010). In the present study, we isolated and characterized three phages, vB_EcoM_SYGD1, vB_EcoP_SYGE1, and vB_EcoM_SYGMH1, with broad host spectrum. The major advantages of phage therapy are specificity and strong bactericidal activity. However, Costa et al. have reported that *E. coli* can develop resistance to phage at a short time within 12 h (Costa et al., 2019). To overcome this disadvantage, phage cocktail comprising multiple phages may be considered an efficacy treatment modality (Costa et al., 2019; Pires et al., 2020). In our study, the three phages showed a high EOP against drug-resistant bacteria (EOP ≥ 0.1), which displayed the same or higher antibacterial efficacy as other reported lytic *E. coli* phage *in vitro* (Tolen et al., 2018; Montso et al., 2019). In addition, they had a broad host spectrum and could lysis *E.coli* strains with different degrees of antibiotic resistance. Compared with the other two phages, SYGE1 could lyse more strains (16 of 20, 80%), which indicated that phage cocktails could lyse a wider range of hosts than a single phage. The phage multiplication stages in a life cycle were divided into attachment, adsorption, injection, biosynthesis, maturation, assembly, and lysis (Yang et al., 2019). The parameters of phage life cycle play a significant role in determining both *in vitro* and *in vivo* phage antibacterial activities, because phage multiplication is directly proportional to reduction in bacteria (Manohar et al., 2019). In this study, SYGD1 had a growth profile with the latent period of 20 min and burst size of 51.7 PFU/cell. SYGE1 and SYGMH1 had a latent period of 10 min which was shorter than earlier studies and also had a high burst size (Dalmasso et al., 2016; Manohar et al., 2019). The three phages had different latent period and higher productive potential indicates they could deliver sufficient infective phages where problematic bacteria appear and achieve the success of microbial control in the form of phage cocktail (Pires et al., 2020).

Understanding the genetic information of phage is essential for the safe and efficient clinical application (Shahin et al., 2020). All three isolated phages lacked harmful genes, such as lysogenic genes, antimicrobial resistance, and bacterial virulence, suggesting that they met the first criterion for phage therapy (Shahin et al., 2020). Moreover, the genomes of all the three phages showed 90% similarities to the already existing phage genomes in the database indicating that there is abundance of these phages in the environment and is valuable to study the interaction of phages and their host for therapeutic purpose (Manohar et al., 2019). Phages may be inactivated by various environmental stresses before reaching the target bacteria (Pires et al., 2020). The stability of phage preparations is a key requirement for successful treatment. In our study, SYGD1, SYGE1, and SYGMH1 were found to be relatively stable at various temperatures, pH values, and chloroform values, meaning that these three phages could be potential phage candidates for therapy.

Extensive clinical research and studies on bacteriophages and phage therapy could put forth phage therapy as one of the alternative treatment against ‘superbug’ infections (Manohar et al., 2019). However, a single phage therapy is easier to develop its resistance cells (Kaabi and Musafer, 2020). So, the phage mixtures could effective to solve this problem. As expected, our results, which was similar to most reported treatment effects of phage cocktails, exhibited that the symptoms of animals have improved significantly (Tanji et al., 2005b; Jin et al., 2020; Nale et al., 2021). Furthermore, the innate inflammatory response is the initial stage of infection and is a key factor in protecting the body from infectious pathogens (Khan et al., 2015). In numerous *in vitro* and *in vivo* studies, proinflammatory signals, such as TNF-α, IL-1β, and IL-6, have been implicated in deviating immune responses to infection with *S. aureus* and *E. coli* (Bannerman et al., 2004). In our study, the cows’ blood had high concentrations of IL-1β and TNF-α when infected with *E. coli* which was consistent with early study (Bannerman et al., 2004), but the release of these proinflammatory mediators dropped to a lower level on the third day after three consecutive treatments with phages or antibiotics. Phage therapy achieved the similar therapeutic effect of antibiotics suggesting that phage cocktails could lysis bacteria effectively *in vivo* and reduce the host inflammation. Moreover, the response of the immune system may be more important to the severity of the disease than the infection itself. The cell wall of lipopolysaccharides (LPS) in *E. coli* is a key virulence factor, which induces the upregulation of pro-inflammatory cytokines during the mastitis (Griesbeck-Zilch et al., 2008 and Guha and Mackman, 2001). Notably, *E. coli*, which makes use of LPS as a receptor of phage, generally evades phage infection by mutating genes involved in LPS biosynthesis (Labrie et al., 2010). This change in structure and function of LPS under the pressure of phage infection is usually accompanied by pleiotropic fitness costs which may have negative consequences (such as decrease in virulence) in pathogenic bacterial populations (Burmeister et al., 2020). Although, in this study, it was difficult to distinguish whether the effect is from bacteriolysis of phage or from decrease in pathogenic bacterial populations as mentioned above played the key role during phage therapy, the treatment with a phage cocktail significantly alleviates the symptoms of mastitis in cows. It is clear that SYGD1, SYGE1, and SYGMH1 in the form of phage cocktails could be the candidates of phage therapy.

## Conclusion

Overall, antibiotic resistance and the transfer of resistant genes should be considered serious public health issues. Phages are a powerful option for the post-antibiotic era. This study described the isolation and characteristics of three phages of *E.coli* and evaluated their therapeutic effects in cow mastitis caused by drug resistant *E.coli*. We found that these three phages show promise as antimicrobial agents especially when used in a cocktail to significantly reduce the number of bacteria, somatic cells, and inflammatory factors, alleviates the symptoms of mastitis in cattle, and achieves the same effect as antibiotic treatment. It enhanced our knowledge about phage information and increased our confidence in using phage cocktails. Further research on the efficacy of phages in therapeutic applications will be significant.

## Data Availability Statement

The whole-genome sequences of vB_EcoM_SYGD1, vB_EcoP_SYGE1, and vB_EcoM_SYGMH1 can be found in NCBI using accession numbers MW883059, MW883060 and MW883061.

## Ethics Statement

The animal study was reviewed and approved by The Ethical Committee for Animal Experiments of Shanghai Jiao Tong University. Written informed consent was obtained from the owners for the participation of their animals in this study.

## Author Contributions

YY and ZW designed the experiments. ZW, MG, and YG performed the experiments and collected the data. MG, YX, YL, XZ, and YC collected and analyzed the data. JS, JM, and HW performed critical revision of the article. MG and ZW wrote the manuscript. All authors contributed to the article and approved the submitted version.

## Funding

This study was funded by the Science and Technology Commission of Shanghai Municipality (18391901900), the National Key Research and Development Program of China (2019YFA0904000), and the National Natural Science Foundation of China (31772744, 32072822, and 31902237).

## Conflict of Interest

The authors declare that the research was conducted in the absence of any commercial or financial relationships that could be construed as a potential conflict of interest.
