# Supplementary material for: Bacteriophage Cocktails Protect Dairy Cows Against Mastitis Caused By Drug Resistant Escherichia coli Infection
Source: Front Cell Infect Microbiol. 2021 Jun 17;11:690377. doi: 10.3389/fcimb.2021.690377 (PMC8248792; doi:10.3389/fcimb.2021.690377)
Supplement: Supplementary file 1 [file Table_1.docx]

**Supplementary Material**

**TABLE S1** The sequence identity of the vB_EcoM_SYGD1 genome with other *Escherichia* *coli* phage

| Accession | Other phages | Phage type | Genome size (bp) | Morphology | Query cover |
| --- | --- | --- | --- | --- | --- |
| MH243439.1 | *Escherichia* phage vB_EcoM_NBG2 | Lytic | 166083 | Myoviridae | 97.03% |
| MH051915.1 | *Enterobacteria* phage vB_EcoM_IME339 | Lytic | 164366 | Myoviridae | 97% |
| MT179807.1 | *Escherichia* phage vB_EcoM_IME537 | Lytic | 168642 | Myoviridae | 96.9% |
| MT682709.1 | *Escherichia* phage vB_EcoM_SP1 | Lytic | 165416 | Myoviridae | 96.89% |
| MH837626.1 | *Escherichia* phage vB_vPM_PD112 | Lytic | 168084 | Myoviridae | 96.79% |

**TABLE S2** The sequence identity of the vB_EcoP_SYGE1 genome with other *Escherichia coli* phage

| Accession | Other phages | Phage type | Genome size (bp) | Morphology | Query cover |
| --- | --- | --- | --- | --- | --- |
| EU734174.1 | *Enterobacteria* phage 13a | Lytic | 38841 | Podoviridae | 93.96% |
| AY264774.1 | *Enterobacteria* phage T7Quinobequin-P09 | Lytic | 39938 | Podoviridae | 93.96% |
| V01146.1 | Genome of bacteriophage T7 | Lytic | 39937 | Podoviridae | 93.96% |
| GU071091.1 | *Enterobacteria* phage T7 | Lytic | 39778 | Podoviridae | 93.94% |
| MW248381.1 | *Escherichia* phage T7 clone T7Syn | Lytic | 39950 | Podoviridae | 93.78% |

**TABLE S3** The sequence identity of the vB_EcoM_SYGMH1 genome with other *Escherichia coli* phage

| Accession | Other phages | Phage type | Genome size (bp) | Morphology | Query cover |
| --- | --- | --- | --- | --- | --- |
| MT944117 | *Escherichia* phage PNJ1809-36 | Lytic | 152343 | Myoviridae | 97.79% |
| JQ031132.1 | *Enterobacteria* phage vB_EcoM-FV3 | Lytic | 136947 | Myoviridae | 96.65% |
| MK883717 | *Eschericha* phage vB_EcoM-ECP26 | Lytic | 136993 | Myoviridae | 95.74% |
| MK962749 | Shigella phage CM1 | Lytic | 139598 | Myoviridae | 95.35% |
| LN881727 | *Escherichia* phage slur16 | Lytic | 136896 | Myoviridae | 95.19% |

**TABLE S4** tRNA coding regions in the genome of vB_EcoM_SYGD1

| tRNA | tRNA Begin | tRNA End | tRNA Type | Anticodon |
| --- | --- | --- | --- | --- |
| 1 | 136906 | 136978 | Gln | TTG |
| 2 | 136980 | 137066 | Leu | TAA |
| 3 | 137073 | 137143 | Gly | TCC |
| 4 | 137157 | 137230 | Pro | TGG |
| 5 | 137233 | 137319 | Ser | TGA |
| 6 | 137327 | 137399 | Thr | TGT |
| 7 | 137406 | 137477 | Ile2 | CAT |
| 8 | 138188 | 138264 | Arg | TCT |

**TABLE S5** tRNA coding regions in the genome of vB_EcoM_SYGMH1

| tRNA | tRNA Begin | tRNA End | tRNA Type | Anticodon |
| --- | --- | --- | --- | --- |
| 1 | 104046 | 104120 | Arg | TCT |
| 2 | 104251 | 104338 | Tyr | GTA |
| 3 | 104642 | 104738 | Lys | TTT |
| 4 | 104745 | 104820 | Thr | TGT |
| 5 | 104822 | 104896 | Ile2 | CAT |
| 6 | 104908 | 104984 | Pro | TGG |
